# Supplementary material for: The economic impact of childhood acute gastroenteritis on Malawian families and the healthcare system
Source: BMJ Open. 2017 Sep 3;7(9):e017347. doi: 10.1136/bmjopen-2017-017347 (PMC5589001; doi:10.1136/bmjopen-2017-017347)
Supplement: Author's manuscript [file bmjopen-2017-017347.draft_revisions.pdf]

BMJ Open is committed to open peer review. As part of this commitment we make the peer review history of every article we publish publicly available.

When an article is published we post the peer reviewers' comments and the authors' responses online. We also post the versions of the paper that were used during peer review. These are the versions that the peer review comments apply to.

The versions of the paper that follow are the versions that were submitted during the peer review process. They are not the versions of record or the final published versions. They should not be cited or distributed as the published version of this manuscript.

BMJ Open is an open access journal and the full, final, typeset and author-corrected version of record of the manuscript is available on our site with no access controls, subscription charges or pay-per-view fees (http://bmjopen.bmj.com).

If you have any questions on BMJ Open's open peer review process please email info.bmjopen@bmj.com

BMJ Open

The economic impact of childhood acute gastroenteritis on Malawian families and the healthcare system

Journal:	*BMJ Open*
Manuscript ID	bmjopen-2017-017347
Article Type:	Research
Date Submitted by the Author:	22-Apr-2017
Complete List of Authors:	Hendrix, Nathaniel; University of Washington, Pharmaceutical Outcomes Research and Policy Program<br>Bar-Zeev, Naor; University of Liverpool, The Centre for Global Vaccine Research, Institute of Infection & Global Health; University of Malawi, Malawi-Liverpool-Wellcome Trust Clinical Research Programme, College of Medicine<br>Atherly, Deborah; PATH<br>Chikafa, Jean; Malawi-Liverpool-Wellcome Trust Clinical Research Programme, College of Medicine, University of Malawi<br>Mvula, Hazzie; Karonga Prevention Study; London School of Hygiene and Tropical Medicine Department of Infectious Disease Epidemiology<br>Wachepa, Richard; Malawi-Liverpool-Wellcome Trust Clinical Research Programme, College of Medicine, University of Malawi<br>Crampin, Amelia; London School of Hygiene and Tropical Medicine Department of Infectious Disease Epidemiology<br>Mhango, Themba; Queen Elizabeth Central Hospital<br>Mwansambo, Charles; Government of Malawi Ministry of Health,<br>Heyderman, Robert; University College London, Division of Infection and Immunity; University of Malawi College of Medicine, Malawi Liverpool Wellcome Trust Clinical Research Programme<br>French, Neil; The Centre for Global Vaccine Research, Institute of Infection & Global Health; Malawi-Liverpool-Wellcome Trust Clinical Research Programme, College of Medicine, University of Malawi<br>Cunliffe, Nigel; University of Liverpool, Clinical Infection, Microbiology and Immunology<br>Pecenka, Clint; PATH
**Primary Subject Heading**:	Global health
Secondary Subject Heading:	Gastroenterology and hepatology, Health economics, Paediatrics
Keywords:	Gastrointestinal infections < GASTROENTEROLOGY, Paediatric gastroenterology < GASTROENTEROLOGY, Health economics < HEALTH SERVICES ADMINISTRATION & MANAGEMENT, International health services < HEALTH SERVICES ADMINISTRATION & MANAGEMENT, Tropical medicine < INFECTIOUS DISEASES

For peer review only

The economic impact of childhood acute gastroenteritis on Malawian families and the healthcare system

Authors: Nathaniel Hendrix[1,2], Naor Bar-Zeev[3,4], Deborah Atherly[1], Jean Chikafa[4], Hazzie Mvula[5,6], Richard Wachepa[4], Amelia C Crampin[5,6], Themba Mhango[7] , Charles Mwansambo[8],Robert S Heyderman[4,9], Neil French[3,4], Nigel A Cunliffe[3], Clint Pecenka[1], for the VacSurv Consortium

Affiliations:
1. PATH, Seattle, USA
2. Pharmaceutical Outcomes Research and Policy Program, University of Washington, Seattle, USA.
3. The Centre for Global Vaccine Research, Institute of Infection & Global Health, University of Liverpool, Liverpool UK
4. Malawi-Liverpool-Wellcome Trust Clinical Research Programme, College of Medicine, University of Malawi, Blantyre Malawi
5. Karonga Prevention Study, Chilumba Malawi
6. London School of Hygiene and Tropical Medicine, London UK
7. Queen Elizabeth Central Hospital, Blantyre, Malawi
8. Ministry of Health, Lilongwe, Malawi
9. Division of Infection & Immunity, University College London, London UK

Corresponding author: Clint Pecenka

Tel: +1-206-285-3500

Email: cpecenka@path.org

Abstract word count: 199; text word count: 3,529

Running title: “Impact of childhood gastroenteritis in Malawi”

Key words: Malawi, gastroenteritis, diarrheal disease, low-income countries, burden of disease, medical impoverishment

For peer review only

ABSTRACT

Objectives: This prospective cohort study sought to estimate health system and household costs for episodes of diarrheal illness in Malawi.

Setting: Data was collected in two Malawian settings: a rural health center in Chilumba, and an urban tertiary care hospital in Blantyre.

Participants: Children under five years of age presenting with diarrheal disease between January 1, 2013 and November 21, 2014 were eligible for inclusion. Illnesses attributed to other underlying causes were excluded, as were illnesses commencing more than two weeks prior to presentation. Complete data was collected on 514 cases at both the time of the initial visit to the participating healthcare facility and six weeks after discharge.

Primary and secondary outcome measures: The primary outcome measure was the total cost of an episode of illness. Costs to the health system were gathered from chart review (drugs and diagnostics) and actual hospital expenditure (staff and facility costs). Household costs, including lost income, were obtained by interview with the parents/guardians of patients.

Results: Total costs in 2014 US$ for rural inpatient, rural outpatient, urban inpatient and urban outpatient were $65.33, $8.89, $60.23, and $14.51, respectively (excluding lost income). Mean household contributions to these costs were 15.8%, 9.8%, 21.3%, and 50.6%.

Conclusion: This study found significant financial burden from childhood diarrheal disease to the healthcare system and to households. The latter face the risk of consequent impoverishment, as the study demonstrates how the costs of seeking treatment bring the

For peer review only - http://bmjopen.bmj.com/site/about/guidelines.xhtml

income of the majority of families in all income strata below the national poverty line in the month of illness.

Strengths and limitations

- We collected costs of illness directly from patient caregivers and clinic records rather than relying on model-derived estimates.
- Collection of information on lost income means that we were able to provide more holistic measures of the effect of illness on household economy.
- Only two clinics were sampled, so the conclusions' generalizability to other locations in Malawi is uncertain.
- Estimates of the contribution of previous visits to overall costs are likely to be confounded by selection bias, as the most severe patients are likely to have been seen before and to have higher costs.

For peer review only

INTRODUCTION

Diarrhea is a major cause of childhood illness and death. It is responsible for an estimated 9% of all under-five deaths globally[1], and in Malawi results in at least 3,000 under-5 deaths annually[2]. Of all pathogens, rotavirus causes the largest share of those deaths, accounting for at least 25%[3]. The toll of diarrheal disease can be mitigated by treatments such as oral rehydration solution, zinc and in some cases, antibiotics or hospital-based care. Vaccines also play an important role in mitigating the spread and severity of diarrheal disease. Rotavirus vaccines, for example, have been introduced in many countries, including Malawi, where they prevent infection and severe disease, and have resulted in substantial reductions in rotavirus-associated illness and death[4,5].

In addition to the burden of disease and death in children, treatment costs can cause significant financial strain to households and health care systems alike. In order to understand comprehensively the impact of diarrheal disease and the potential economic benefit of vaccines, one must examine the actual costs of care incurred by the health care system and by households.

Most economic evaluations have relied on model-derived cost estimates based on extant, retrospective data. Very few studies have prospectively collected direct individual patient-level costs of diarrheal illness at both inpatient and outpatient settings[6]. Even fewer have collected such data from health facilities and households, including direct and indirect medical costs and income loss estimates[7]. Additionally, many studies lack data on household income that would permit examination of differential short-term financial effects of illness by income level.

For peer review only - http://bmjopen.bmj.com/site/about/guidelines.xhtml

In this post-hoc analysis of data collected for a cost-effectiveness analysis[8], we sought to provide insight into the cost of treating diarrheal disease from the health system and societal perspectives in the context of a low-income country where receipt of both doses of the rotavirus vaccine is near 90%[9]. In other low-income settings, geographic setting (urban/rural), treatment setting (inpatient/outpatient), household income, and duration of illness have consistently demonstrated correlation with the economic burden of diarrheal disease[6,10,11]. We sought to explore these factors that drive cost of illness in this sub-Saharan, low-income setting, as well as to leverage our capture of prior costs to examine the effects of multiple sessions of care on cost.

CONTEXT AND METHODS

Study area and population

This prospective cohort study was conducted in Malawi, a country of approximately 16 million people in southern Africa. Its per capita gross national income of US$350 places it 210th out of 216 countries and territories ranked by the World Bank[12]. Over 80% of its population is rural, with much of that population engaged in subsistence farming[13].

The study was conducted at two sites: Queen Elizabeth Central Hospital (QECH), a national referral hospital that provides both inpatient and outpatient care, and Chilumba Rural Hospital (CRH), which provides outpatient primary care for children under 5 and accepts inpatients, although generally low acuity cases. QECH is located in Blantyre, a district of approximately 1.3 million in Malawi's Southern Region. It receives approximately 90,000 presentations per year, 25,000 of which are admitted to the inpatient facility. CRH is located in Karonga, a district of

For peer review only - http://bmjopen.bmj.com/site/about/guidelines.xhtml

approximately 200,000 in the Northern Region. Chilumba hosts a demographic surveillance site with a population approaching 40,000[14]. It sees 7,500 outpatients per year, of which about 700 are admitted inpatient. Both sites provide inpatient and outpatient care free of charge and were purposively selected to be representative of the care provided at different levels of the health system[8].

Inclusion criteria

Eligible participants were children under five years of age living in Blantyre district or within the demographic surveillance population in Chilumba and presenting with diarrhea at either facility during the study period (January 1, 2013 to November 21, 2014). Illnesses attributed to other underlying causes were excluded, as were illnesses commencing more than two weeks prior to presentation. In addition to identifying participants at registration/triage, admission logs and electronic records (at QECH only) were reviewed for missing cases.

Data collection

After identifying each participant and obtaining consent from their caregiver, a trained research assistant administered a standard case report form to the caregiver. The questionnaire included demographic information, socioeconomic status, and the history of the child's illness prior to presentation at the facility, as well as all illness-associated costs incurred prior to presenting at the facility. Daily review of medical charts was undertaken during admission for inpatients to gather information on drugs dispensed, and laboratory investigations or procedures performed. All participants' homes were visited by research staff six weeks post-

For peer review only - http://bmjopen.bmj.com/site/about/guidelines.xhtml

discharge to evaluate whether any subsequent illness-related costs were incurred by the household.

Categorization of expenses

Pre-treatment costs included direct medical costs paid by the household for care sought prior to the date of the index visit, which we defined as the date of an outpatient visit at either facility or the first day of an inpatient stay. Since the health facilities at which previous care was sought were not study sites, costs to the health system were not included in the pre-index costs.

Costs of the index visit were divided into health system and household costs. Costs to the health system included drugs, laboratory investigations, staff, and facility costs such as laundry, kitchen, sanitation, and security. The cost of drugs was calculated based on acquisition costs for drugs used at the index visit, with the quantity of injectable drugs being rounded up to the nearest full vial size. Investigations performed were costed on charge per test, except for those performed as part of clinical research, which were excluded. Staff costs for inpatient visits used the combined daily salary of all staff present divided by the number of beds in the ward, then multiplied by the patient's length of stay in days; staff costs of emergency outpatient visits were calculated by dividing the combined daily salary of staff by the mean daily number of visits to the department. Facility costs were calculated by dividing the facility's mean daily cost for services and subsistence goods by the number of hospital beds, then multiplying by the patient's length of stay. The cost of consumable goods in the clinic such as tubing for intravenous drips were not included. Household costs included transportation to the index visit

For peer review only - http://bmjopen.bmj.com/site/about/guidelines.xhtml

148 for the patient, their main caretaker and any other household visitors during admission; direct medical costs (e.g., consultation fees, drug costs); lost income attributable to the episode of disease; and subsistence (food and shelter) during the visit. Lost income was calculated by dividing the self-reported monthly income by the number of days in the month, then multiplying by the number of days spent in care-taking for this episode of illness.

153 Ongoing costs were assessed at a follow-up visit to the patient's home six weeks after facility discharge. Costs related to any long-term disability caused by the episode were not included among these ongoing costs. Again, costs to the health system for further treatment could not be ascertained due to other sites' being outside the network of research sites.

157 All costs were collected in Malawi kwacha and converted to US dollars based on the mid-market exchange rate per the Malawi Reserve Bank on July 15, 2014.

159 *Statistical analysis*

160 The study's minimum sample size of 88 was calculated to provide an estimate of household costs with a margin of error ≤10% given a coefficient of variation of 0.5[8,15].

162 Income quintiles were calculated based on self-reported monthly income within the sample itself---that is to say that a household labelled as belonging in the highest income quintile is within the top 20% of household incomes in this sample. Using one-way ANOVA, we tested for differences among income quintiles in mean overall household costs at each geographic and treatment setting.

For peer review only - http://bmjopen.bmj.com/site/about/guidelines.xhtml

To explore associations proposed in previous research between prior treatment and cost, we constructed a generalized linear model (GLM) of total costs and household costs with log link on gamma distributional family, adjusted for length of stay, geographic setting, and treatment setting. We used a chi-squared test to compare the proportion of patients with prior/ongoing costs in different settings, and a rank-sum test to assess differences in component costs between these groups. We also used rank-sum tests to assess the significance of differences in total (health system plus household) and household cost between geographic and treatment settings.

Ethics

Ethical approval was provided by the National Health Sciences Research Committee, Lilongwe, Malawi (1073), and by the Research Ethics Committee of the University of Liverpool, United Kingdom (000490). Written consent was obtained from the parents or guardians of participating children.

RESULTS

Participants

A total of 529 individuals participated in this study, although 15 were excluded due to incomplete data. The majority (389) were recruited at QECH in Blantyre and the remaining 130 participants were recruited at CRH in Karonga. Inpatient cases accounted for 69% (269/389) of the cases at QECH while only 17% (22/130) of cases in Karonga were admitted, reflecting a more serious case mix at a much larger national referral level hospital in Blantyre.

For peer review only - http://bmjopen.bmj.com/site/about/guidelines.xhtml

Table 1: Patient characteristics

	Rural		Urban		
	Inpatient	Outpatient	Inpatient	Outpatient	Total
N (% of total)	22 (4%)	108 (21%)	269 (52%)	120 (23%)	519
Mean Vesikari score (sd)	6.0 (2.9)	2.6 (2.2)	11.1 (3.9)	2.0 (3.4)	7.0 (5.5)
Sex: Male, not recorded(%)	13 (59%), 0	67 (62%), 2 (2%)	159 (59%), 2 (1%)	62 (52%), 1 (1%)	301(58%), 5(1%)
Mean age, months (sd)	14.0 (12.7)	15.8 (11.3)	13.4 (7.4)	15.8 (11.8)	14.3 (9.3)
Median length of stay, days (IQR)	2 (2.5)	N/A	3 (2)	N/A	3 (2) [inpatients only]

Total costs for the episode of illness as well as costs by payer are reported in **Table 2**, sorted by geography and treatment setting. The least expensive visit type was a rural, outpatient visit with total costs averaging $13.57; rural, inpatient visits were the costliest, averaging $76.94. Rank-sum tests confirmed the significance of differences in both total and household costs between geographic and treatment settings (p < 0.001 in all cases). Across all settings, the health system bore an average of 76% of the costs for each episode of illness.

We performed a sensitivity analysis to assess the potential impact to overall costs at the index visit if the 15 dropped observations were included. Were these costs representative of the 25th percentile of costs, mean overall costs would decrease from $39.15 to $38.32; were they in the 75th percentile of costs, mean overall costs would rise to $39.44. This would result in a 2% decrease in total costs, or a 0.6% increase, respectively, compared to the weighted average of costs across all visit types.

Table 2: Mean (SD) total direct and indirect costs for the episode of illness by geography and treatment setting

	Rural	Urban

For peer review only - http://bmjopen.bmj.com/site/about/guidelines.xhtml

	Inpatient	Outpatient	Inpatient	Outpatient
Health system costs	$55.04 ($26.82)	$8.02 ($2.90)	$47.21 ($56.69)	$7.18 ($4.05)
Household costs	$19.16 ($19.30)	$1.81 ($2.58)	$25.36 ($20.47)	$15.48 ($20.75)
Total costs	$76.94 ($32.61)	$13.57 ($4.70)	$73.78 ($51.39)	$23.13 ($21.68)

For inpatient settings, staff and facility costs contribute the most to health system costs (see **Table 3**). These two categories represent between 60 and 80% of total costs across geographic setting. Laboratory investigations dominate outpatient costs, representing roughly 60% in both urban and rural outpatient settings.

Table 3: Mean (SD) costs to health system for index visit by category

	Rural		Urban	
	Inpatient	Outpatient	Inpatient	Outpatient
Drugs	$4.46 ($3.94)	$1.45 ($2.53)	$9.40 ($20.29)	$1.19 ($2.16)
Labs	$5.39 ($1.07)	$4.96 ($1.17)	$7.60 ($5.21)	$4.73 ($3.36)
Staff	$17.03 ($10.37)	$1.61 ($0)	$21.47 ($30.51)	$1.26 ($0)
Facilities	$28.35 ($17.26)	$0 ($0)	$8.73 ($12.41)	$0 ($0)
Total	$55.04 ($26.82)	$8.02 ($2.90)	$47.21 ($56.69)	$7.18 ($4.05)

Lost income and direct medical expenses make up the majority of household costs for all except rural, outpatient visits, whose costs are dominated by transportation and lost income (see **Table 4**). Of patients seen at the urban facility, 35% had sought treatment prior to their index visit, compared to 2% of patients seen at the rural facility ($p < 0.001$). On the other hand, 13%

For peer review only - http://bmjopen.bmj.com/site/about/guidelines.xhtml

212 of patients in Karonga had ongoing costs after the index visit, compared to 2% of patients seen at the Blantyre site ($p < 0.001$).

214 *Table 4: Mean (SD) household costs for an episode of illness*

	Rural		Urban		Total
	Inpatient	Outpatient	Inpatient	Outpatient	
Direct medical expenses prior to index visit	$0.21 ($0.72)	$0.08 ($0.58)	$0.98 ($3.92)	$0.71 ($1.55)	$0.77 ($3.13)
Direct medical expenses of index visit	$8.61 ($9.62)	$0.24 ($0.68)	$10.69 ($10.66)	$5.85 ($10.21)	$7.31 ($10.17)
Transportation to and subsistence at index visit	$4.38 ($5.28)	$0.51 ($0.54)	$2.68 ($3.00)	$0.45 ($1.91)	$2.04 ($2.83)
Lost income due to index visit	$4.13 ($9.62)	$0.50 ($0.68)	$8.06 ($10.66)	$6.22 ($10.22)	$6.36 ($10.17)
Direct medical expenses after index visit	$0.87 ($2.10)	$0.38 ($1.66)	$0.21 ($2.41)	$0.16 ($1.19)	$0.26 ($2.02)
Total household costs	$19.16 ($19.30)	$1.81 ($2.58)	$25.36 ($20.47)	$15.48 ($20.75)	$19.32 ($20.66)

215 Using one-way ANOVA to detect differences in mean household expenditure (exclusive of lost income) between income quintiles, we found a significant difference only in urban, inpatient encounters (see **Figure 1**). The highest income level in this group (> $79.50 per month) had higher expenditures than any other income group, with the difference between this group and the lowest income group (< $19.32 per month) being especially noticeable. The ANOVA test showed no significant differences for any other combination of geographic and treatment settings.

For peer review only - http://bmjopen.bmj.com/site/about/guidelines.xhtml

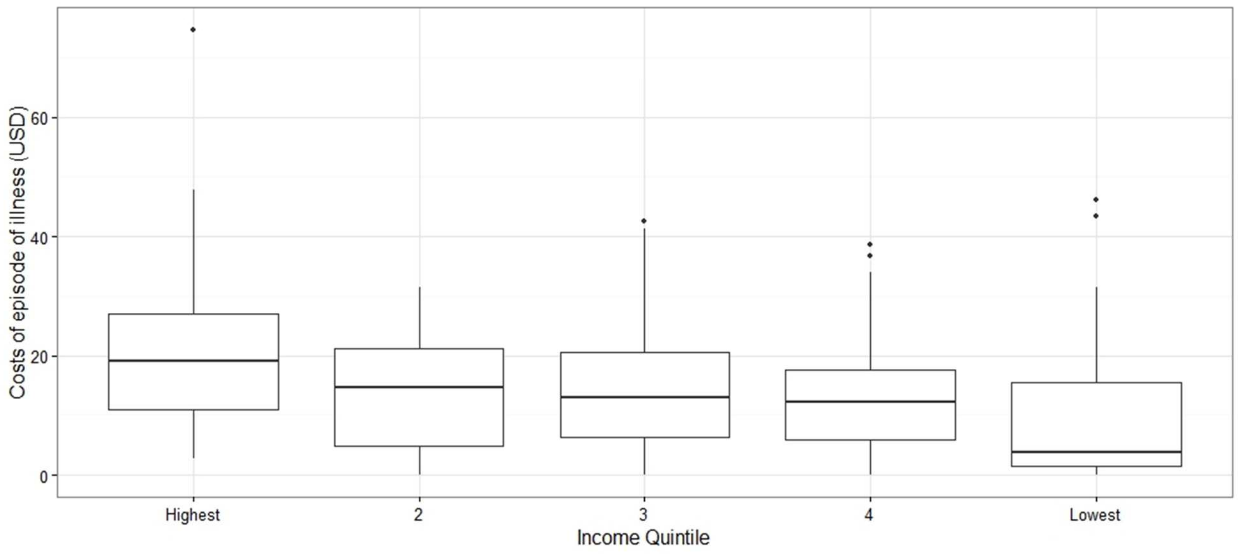

Figure 1: Mean total household cost (not including lost income) of inpatient, urban index visit by income quintile

peer review only

Costs to the households of patients for whom previous treatment was sought were 2.04 times higher ($p < 0.001$) once adjusted for length of stay, geography and treatment setting; overall costs were 1.25 times higher ($p = 0.016$). This difference in overall costs is driven in part by spending prior to the index visit, which averaged between $0.92 for rural, outpatient index visits to $3.01 for rural, inpatient index visits. A rank-sum test on transportation costs by prior treatment status showed significantly higher transportation costs for this group ($p < 0.001$), suggesting the source of these differences. Median monthly income for the group who sought prior treatment was higher than the group who did not, at $34.09 and $22.73, respectively. A rank-sum test again showed a significant difference in both monthly income and lost income due to the illness (length of stay was not significantly different between the groups). We repeated the rank-sum test looking for variation in transportation costs during the rainy seasons (November through April) of those who received prior treatment, but this test's results were not significant.

For peer review only - http://bmjopen.bmj.com/site/about/guidelines.xhtml

237 **DISCUSSION**

238 Even following the introduction of the rotavirus vaccine, diarrheal disease continues to be a significant burden on the world and on low-income countries in particular. While deaths among African children due to diarrhea declined 43% between 2000 and 2013[16], the number of cases remains high, with one study estimating 3.3 episodes of diarrhea annually per child in Africa[17].

243 This study focused on the economic burden of these diseases by combining costs ascertained through both interviews and chart review. It found significant household costs primarily in the inpatient and urban settings with lost income and direct medical costs dominating the contributing costs. Even rural, outpatient visits can be financially problematic, however, if they are frequent, or if the family is especially vulnerable. Household costs for the episode of illness (excluding lost income) exceeded monthly household income in 8% of cases treated inpatient and 3% of cases treated outpatient; including lost income, these numbers rise to 17% and 9%, respectively.

251 Taking into account lost income, mean household costs for patients seen in the rural health center were $19.16 and $1.81 for inpatient and outpatient treatment, while patients seen at the urban hospital had mean household costs of $25.36 and $15.48 for inpatient and outpatient treatment. The high cost of inpatient treatment in the urban setting likely reflects the higher acuity of illness at that setting: the mean Vesikari score of study participants seen in QECH's inpatient ward was 11, which indicates severe disease. Rural inpatients, by comparison, had a mean Vesikari score of 6.

For peer review only - http://bmjopen.bmj.com/site/about/guidelines.xhtml

Putting household expenditures in context, 72.2% of the Malawian population lives on less than $1.25 per person per day[18]. Even an outpatient visit in a rural setting, then, is likely to impose costs exceeding a day's income for this population, while a costlier inpatient visit is likely to account for almost 3 weeks' income. The equivalents for a US household would be to face costs of $147 and $3,095, respectively[19]. It should be noted, though, that in a low-income setting, a much larger percent of income goes to core goods such as food and shelter: as such, it is more difficult to offset health-related costs. Having savings adequate to cover medical costs is also less common in this setting, where, although 76% report saving when possible, 90% of households say that they sometimes run out of money for basic goods[20]. This can bring about debt and reduced expenditure on clothing, education, shelter, and other similar goods.

The results of our study can be compared with several other studies of the economic burden of diarrheal disease in the low-income, sub-Saharan context. An analysis of economic data from the Global Multicenter Enteric Study found mean household costs of $2.77, $6.57, and $4.33 (in 2014 USD) for Gambia, Kenya, and Mali, respectively, for a representative sample of inpatient and outpatient medical encounters, not including lost income[21]. A Rwandan study of inpatient admissions for childhood gastroenteritis found household costs of $66.08 (including lost income) and costs to the medical system of $34.83[22]. Finally, a study from Ghana found costs to the medical system of $5.14 for outpatient visits and $122.07 for inpatient visits[7].

When costs are analyzed by income quintile, mean household expenditure by quintile is only significantly different in the urban, inpatient setting. This demonstrates that the poorest segments of Malawian society who attend public healthcare facilities generally incur the same

For peer review only - http://bmjopen.bmj.com/site/about/guidelines.xhtml

costs as their wealthier peers in the same settings. This finding is supported by other evidence from Malawi which found that care-seeking behavior for children with diarrhea does not vary by income level[23].

The pattern of identical expenditure across income quintiles in most settings means that the poorest households expose themselves to greater risk of impoverishment by seeking treatment (see **Figure 2**). After accounting for the costs of the episode of illness, no household in the lowest income quintile of our sample was left with more than $0.60 of income per day for the month in which the illness occurred—well below the government's definition of "ultra-poverty" as $1.74 per household per day[24]. Furthermore, the costs of the episode of illness exceeded the monthly income of 27% of households in this group. Even among the highest income quintile of our sample, 19% of households were brought temporarily below the poverty line of $2.80 per day by the costs of the episode of illness.

For peer review only

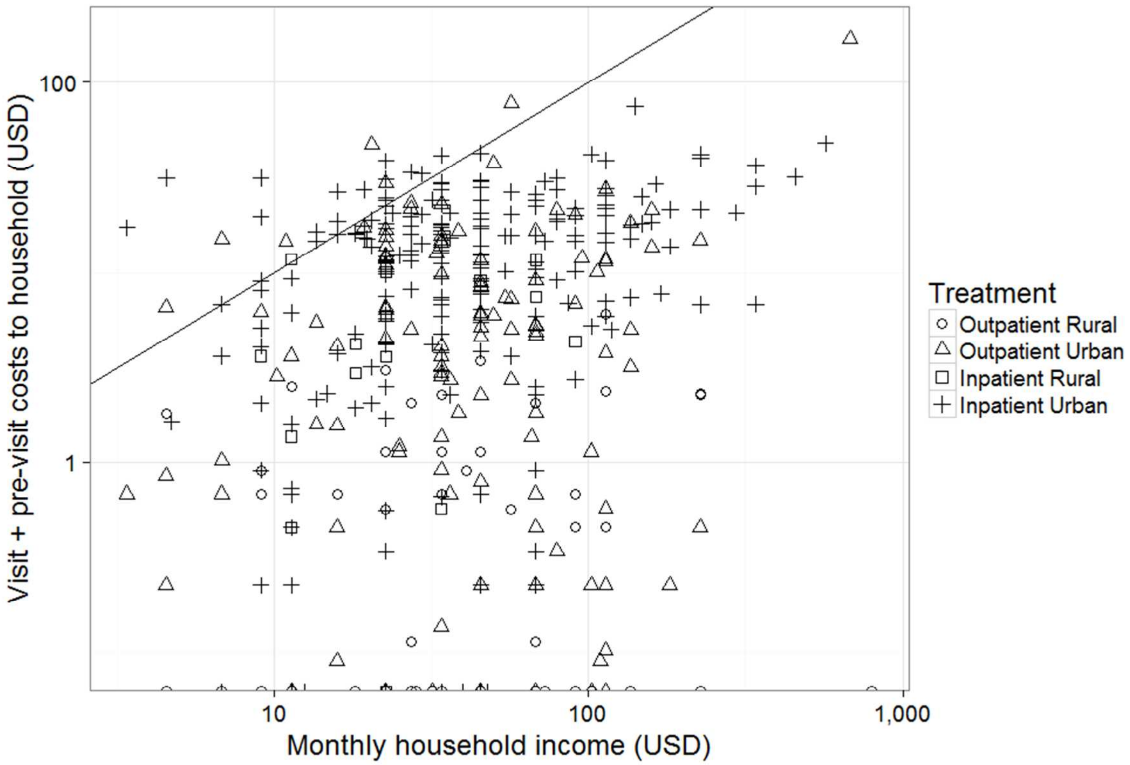

Figure 2: Log monthly household income versus log costs for the episode of illness, not including lost income. The line represents episode costs equal to monthly income.

review only

We are left to explain why spending increases with income quintile in the urban inpatient setting (see **Figure 1**). One explanation may be that price sensitivity may play a role in care decisions in the urban inpatient setting. If this were the case, the children of poorer Malawians may not receive the full extent of care that they need due to concerns about the costs associated with seeking treatment, even when the treatment itself is free. Mean total household costs were the highest for this setting, and the patients' caretakers must balance the risks of expenses that they cannot afford with the risk that their child's illness will worsen when they choose to seek or not seek care. Lower income families may, rationally, apply a different threshold for seeking treatment than higher income families. Another possibility may be a

For peer review only - http://bmjopen.bmj.com/site/about/guidelines.xhtml

tendency of higher income families to bring more visitors or to buy higher cost subsistence goods. In order to test this possibility, we ran an ANOVA test on mean spending by income quintile on visitors, transportation, and shelter costs (both together and separately), but did not find significant differences. Instead, the same test shows significantly increased mean spending on direct medical expenses as income quintile increases.

One driver of high overall costs in the urban setting at large may be the seeking of prior care. Of patients seen in the urban setting, 35% had been seen at a prior healthcare facility. Testing the relationship between prior care and income quintile using a logistic regression does not suggest a significant relationship between the two, whether income quintile is represented as a continuous or factor variable. Excluding lost wages, household costs for cases in which prior care is sought cost twice as much as cases in which the index visit is the first encounter with the healthcare system during this episode of illness. Transportation costs are the likely source of difference, suggesting that patients who sought treatment prior to the index visit come to the hospital either from farther away or more urgently than those for whom the index visit was their first visit related to this episode. Selection bias likely plays a role as well, as the most seriously ill patients are likely both to be seen multiple times and to incur greater overall costs.

The conclusions in this study are limited by its being a post-hoc, unpowered analysis of data gathered for a cost-effectiveness study. That is, the sample size of the study was not powered to detect differences between treatment settings, geography, or any of the other characteristics examined in our regression analysis. The study's generalizability may also be limited due to the data collection's having taken place in two locations that represent extremes

For peer review only - http://bmjopen.bmj.com/site/about/guidelines.xhtml

of acuity and population density within the Malawian healthcare system. We cannot rule out greater geographic variation in financial outcomes than our study results suggest.

Future work in this area should focus on the long-term consequences to the household of financial shock associated with an episode of diarrheal disease, or with repeated diarrhea due to undernourishment and other risk factors. Especially of interest is the long-term loss of economic potential due to childhood illness: if a child is frequently or severely ill, health-related expenditures, including time needed for caretaking, may preclude investment in other areas that could improve the financial situation of a household.

Conclusion

This study has evaluated directly the cost of 514 episodes of childhood diarrheal disease to both the households and health system of Malawi. It found that households bear a substantial fraction of the total costs of an episode of illness and that there is a considerable risk of impoverishment due to severe or repeated episodes of diarrheal disease.

The conclusions of this study are broadly applicable in the sub-Saharan setting—especially to those countries where the rotavirus vaccine is widely used—regardless of whether healthcare is provided to the public for free or through an insurance program. In the latter case, only direct medical costs to the household would be covered. Lost income, transportation, and ongoing costs of care were all found in this study to impose substantial financial burdens, and this observation would not be changed by the use of health insurance.

For peer review only

For peer review only - http://bmjopen.bmj.com/site/about/guidelines.xhtml

343 This study's findings can be applied to cost-effectiveness analyses of enteric vaccines and other means of preventing the transmission of diarrheal disease and can be used by healthcare planners to help prioritize interventions. By providing data on the lost financial opportunity caused by an episode of diarrheal disease, this study allows for a more precise accounting of how an averted episode of diarrheal disease should be valued.

For peer review only

For peer review only - http://bmjopen.bmj.com/site/about/guidelines.xhtml

Conflict of interest: NBZ, NAC and NF have received investigator-initiated research grants from GSK; and NBZ and NAC from Takeda Pharmaceuticals. All other authors have no conflicts to declare.

Contributorship statement: NB-Z, DA, and NAC designed the study with input from AC, RSH, and NF. NB-Z, JC, HM, RW, CM, and TM obtained the data. NH, NB-Z, CP, and DA designed and performed the data analysis. NH wrote the first draft of the manuscript. All listed authors contributed to the interpretation of the data, reviewed the work prior to submission, and have agreed to be responsible for its content.

Data sharing statement: Data collected as part of this study is not available due to its not having been included in the approved protocol.

Funding: This work was supported by Wellcome Trust Programme Grant [WT091909], PATH and the Bill & Melinda Gates Foundation [OPP1053539].

Preliminary results from this study were presented at the 12th International Rotavirus Symposium in Melbourne, Victoria.

Corresponding author: Clint Pecenka

Tel: +1-206-285-3500

Email: cpecenka@path.org

Members of the VacSurv Consortium:

For peer review only

James Beard (London School of Hygiene and Tropical Medicine [LSHTM], London, UK); Anthony Costello (World Health Organization, Geneva Switzerland; formerly University College London [UCL], London, UK); Miren Iturriza-Gomara (University of Liverpool [UoL], Liverpool, UK); Khuzwayo Jere (UoL) Carina King (UCL, London, UK); Sonia Lewycka (University of Auckland, Auckland, New Zealand; formerly UCL); Osamu Nakagomi (Nagasaki University, Japan); Umesh Parashar (Centers for Disease Control & Prevention [CDC], Atlanta, GA, USA); Tambosi Phiri (Mai Mwana Project, Mchinji, Malawi); Jacqueline E Tate (CDC) Atlanta, GA, USA; Jennifer R Verani (CDC) Atlanta, GA, USA; Cynthia G Whitney (CDC) Atlanta, GA, USA.

References

1. Liu L, Johnson H, Cousens S. Global, regional, and national causes of child mortality in 2000-2010: an updated systematic analysis. Under Revis with Lancet [Internet]. Elsevier Ltd; **2012**; 6736(14). Available from: http://dx.doi.org/10.1016/S0140-6736(14)61698-6

2. World Health Organization. Global Health Observatory Data Repository [Internet]. WHO. World Health Organization; 2016 [cited 2016 Aug 3]. Available from: http://www.who.int/gho/database/en/

3. Institute for Health Metrics and Evaluation. The Global Burden of Disease: Generating Evidence, Guiding Policy [Internet]. Seattle, WA; 2013. Available from: http://www.healthdata.org/policy-report/global-burden-disease-generating-evidence-guiding-policy

For peer review only

387 4. Bar-Zeev N, Kapanda L, Tate JE, et al. Effectiveness of a monovalent rotavirus vaccine in infants in Malawi after programmatic roll-out: an observational and case-control study. Lancet Infect Dis [Internet]. **2015** [cited 2016 Nov 5]; 15(4):422–428. Available from: http://linkinghub.elsevier.com/retrieve/pii/S1473309914710606

391 5. Groome MJ, Page N, Cortese MM, et al. Effectiveness of monovalent human rotavirus vaccine against admission to hospital for acute rotavirus diarrhoea in South African children: a case-control study. Lancet Infect Dis [Internet]. **2014** [cited 2016 Nov 5]; 14(11):1096–1104. Available from: http://linkinghub.elsevier.com/retrieve/pii/S1473309914709405

396 6. Rheingans R, Kukla M, Faruque ASG, et al. Determinants of household costs associated with childhood diarrhea in 3 South Asian settings. Clin Infect Dis [Internet]. **2012** [cited 2016 Aug 3]; 55 Suppl 4:S327-35. Available from: http://www.ncbi.nlm.nih.gov/pubmed/23169945

400 7. Aikins M, Armah G, Akazili J, Hodgson A. Hospital health care cost of diarrheal disease in Northern Ghana. J Infect Dis [Internet]. **2010** [cited 2016 Aug 3]; 202 Suppl:S126-30. Available from: http://www.ncbi.nlm.nih.gov/pubmed/20684692

403 8. Bar-Zeev N, Tate JE, Pecenka C, et al. Cost-Effectiveness of Monovalent Rotavirus Vaccination of Infants in Malawi: A Postintroduction Analysis Using Individual Patient–Level Costing Data. Clin Infect Dis [Internet]. **2016** [cited 2016 Nov 5]; 62(suppl 2):S220–S228. Available from: http://cid.oxfordjournals.org/lookup/doi/10.1093/cid/civ1025

For peer review only - http://bmjopen.bmj.com/site/about/guidelines.xhtml

9. Bar-Zeev N, Kapanda L, Tate JE, et al. Effectiveness of a monovalent rotavirus vaccine in infants in Malawi after programmatic roll-out: an observational and case-control study. Lancet Infect Dis [Internet]. **2015** [cited 2016 Nov 5]; 15(4):422–8. Available from: http://www.ncbi.nlm.nih.gov/pubmed/25638521

10. Burke RM, Rebolledo PA, Embrey SR, et al. The burden of pediatric diarrhea: a cross-sectional study of incurred costs and perceptions of cost among Bolivian families. BMC Public Health [Internet]. BioMed Central; **2013** [cited 2016 Dec 2]; 13(1):708. Available from: http://bmcpublichealth.biomedcentral.com/articles/10.1186/1471-2458-13-708

11. MacIntyre UE, Villiers FPR de. The economic burden of diarrheal disease in a tertiary level hospital, Gauteng, South Africa. J Infect Dis [Internet]. Oxford University Press; **2010** [cited 2016 Dec 2]; 202 Suppl(Supplement 1):S116-25. Available from: http://www.ncbi.nlm.nih.gov/pubmed/20684690

12. World Bank. Gross National Income Per Capita 2015 (Atlas Method) [Internet]. [cited 2016 Nov 5]. Available from: http://databank.worldbank.org/data/download/GNIPC.pdf

13. National Statistical Office [Malawi], ICF International. Malawi Demographic and Health Survey. Zomba, Malawi and Rockville, Maryland, USA; 2015.

14. Crampin AC, Dube A, Mboma S, et al. Profile: the Karonga Health and Demographic Surveillance System. Int J Epidemiol [Internet]. **2012** [cited 2016 Aug 3]; 41(3):676–85. Available from: http://www.ncbi.nlm.nih.gov/pubmed/22729235

15. World Health Organization. Guidelines for estimating the economic burden of diarrhoeal

For peer review only

For peer review only - http://bmjopen.bmj.com/site/about/guidelines.xhtml

disease with focus on assessing the costs of rotavirus diarrhoea. Geneva; 2005.

16. Liu L, Oza S, Hogan D, et al. Global, regional, and national causes of child mortality in 2000–13, with projections to inform post-2015 priorities: an updated systematic analysis. Lancet. **2015**; 385(9966):430–440.

17. Fischer Walker CL, Perin J, Aryee MJ, et al. Diarrhea incidence in low- and middle-income countries in 1990 and 2010: a systematic review. BMC Public Health [Internet]. BioMed Central; **2012** [cited 2016 Aug 3]; 12(1):220. Available from: http://bmcpublichealth.biomedcentral.com/articles/10.1186/1471-2458-12-220

18. United Nations Development Programme. Global 2015 Human Development Report. New York, New York, USA; 2015.

19. US. Bureau of the Census, Real Median Household Income in the United States [MEHOINUSA672N] [Internet]. [cited 2016 Aug 28]. Available from: https://fred.stlouisfed.org/series/MEHOINUSA672N

20. Reserve Bank of Malawi. Malawi: Baseline Financial Literacy and Consumer Protection Household Survey. Zomba, Malawi; 2014.

21. Rheingans R, Kukla M, Adegbola RA, et al. Exploring household economic impacts of childhood diarrheal illnesses in 3 African settings. Clin Infect Dis [Internet]. Oxford University Press; **2012** [cited 2017 Jan 1]; 55 Suppl 4(Suppl 4):S317-26. Available from: http://www.ncbi.nlm.nih.gov/pubmed/23169944

22. Ngabo F, Mvundura M, Gazley L, et al. The Economic Burden Attributable to a Child's

For peer review only

For peer review only - http://bmjopen.bmj.com/site/about/guidelines.xhtml

Inpatient Admission for Diarrheal Disease in Rwanda. Griffiths UK, editor. PLoS One [Internet]. Public Library of Science; **2016** [cited 2017 Jan 1]; 11(2):e0149805. Available from: http://dx.plos.org/10.1371/journal.pone.0149805

23. National Statistical Office (NSO) [Malawi] and ICF International. Malawi Demographic and Health Survey 2015-16: Key Indicators Report. Zomba, Malawi, and Rockville, Maryland, USA; 2016.

24. National Statistical Office (NSO) [Malawi]. Integrated Household Survey, 2010-2011. Zomba, Malawi; 2012.

For peer review only

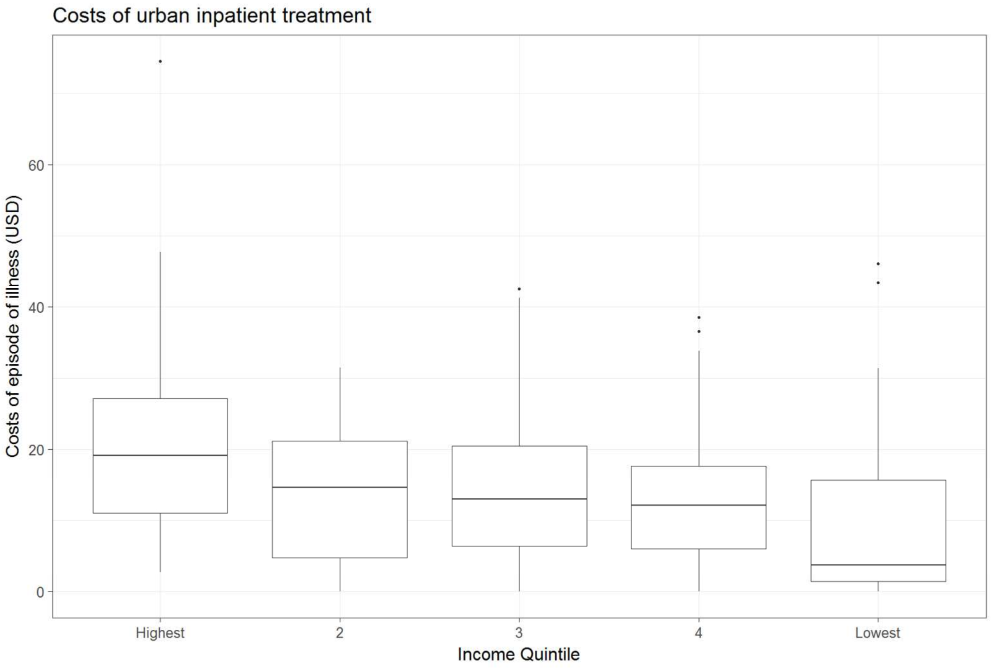

Figure 1: Mean total household cost (not including lost income) of inpatient, urban index visit by income quintile

476x319mm (96 x 96 DPI)

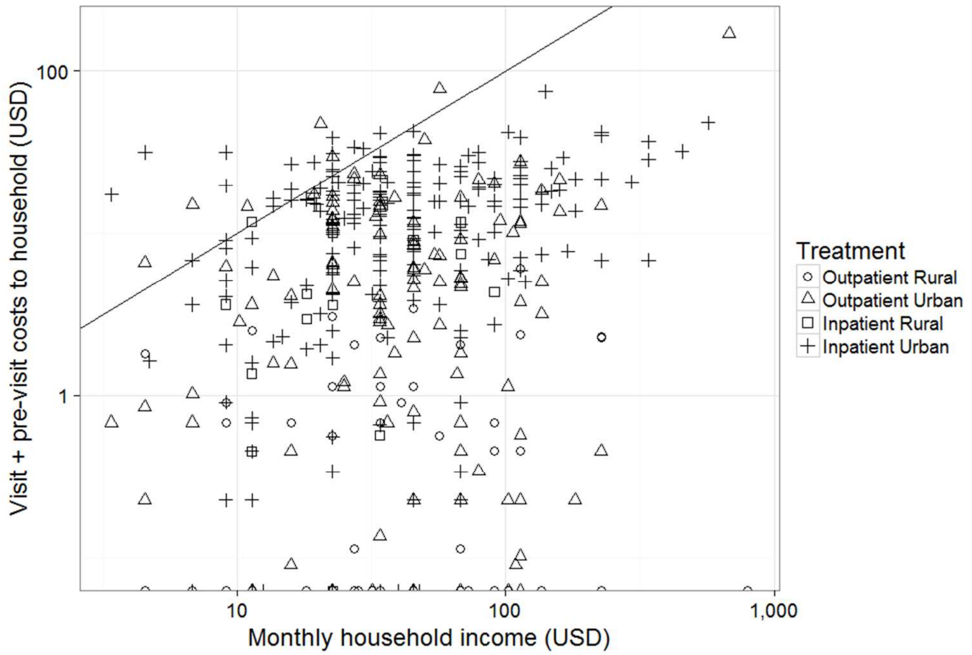

Figure 2: Log monthly household income versus log costs for the episode of illness, not including lost income. The line represents episode costs equal to monthly income.

264x176mm (96 x 96 DPI)

STROBE 2007 (v4) Statement—Checklist of items that should be included in reports of *cohort studies*

Section/Topic	Item #	Recommendation	Reported on page #
**Title and abstract**	1	(*a*) Indicate the study's design with a commonly used term in the title or the abstract	2
		(*b*) Provide in the abstract an informative and balanced summary of what was done and what was found	2
**Introduction**			
Background/rationale	2	Explain the scientific background and rationale for the investigation being reported	4
Objectives	3	State specific objectives, including any prespecified hypotheses	4-5
**Methods**			
Study design	4	Present key elements of study design early in the paper	5
Setting	5	Describe the setting, locations, and relevant dates, including periods of recruitment, exposure, follow-up, and data collection	5-6
Participants	6	(*a*) Give the eligibility criteria, and the sources and methods of selection of participants. Describe methods of follow-up	5-6
		(*b*) For matched studies, give matching criteria and number of exposed and unexposed	N/A
Variables	7	Clearly define all outcomes, exposures, predictors, potential confounders, and effect modifiers. Give diagnostic criteria, if applicable	5-8
Data sources/ measurement	8*	For each variable of interest, give sources of data and details of methods of assessment (measurement). Describe comparability of assessment methods if there is more than one group	7-8
Bias	9	Describe any efforts to address potential sources of bias	17-18
Study size	10	Explain how the study size was arrived at	8
Quantitative variables	11	Explain how quantitative variables were handled in the analyses. If applicable, describe which groupings were chosen and why	6-8
Statistical methods	12	(*a*) Describe all statistical methods, including those used to control for confounding	8-9
		(*b*) Describe any methods used to examine subgroups and interactions	8-9
		(*c*) Explain how missing data were addressed	9-10
		(*d*) If applicable, explain how loss to follow-up was addressed	N/A
		(*e*) Describe any sensitivity analyses	10
**Results**			

For peer review only

Participants	13*	(a) Report numbers of individuals at each stage of study—eg numbers potentially eligible, examined for eligibility, confirmed eligible, included in the study, completing follow-up, and analysed	9
		(b) Give reasons for non-participation at each stage	N/A
		(c) Consider use of a flow diagram	N/A
Descriptive data	14*	(a) Give characteristics of study participants (eg demographic, clinical, social) and information on exposures and potential confounders	9-11
		(b) Indicate number of participants with missing data for each variable of interest	9
		(c) Summarise follow-up time (eg, average and total amount)	6
Outcome data	15*	Report numbers of outcome events or summary measures over time	10
Main results	16	(*a*) Give unadjusted estimates and, if applicable, confounder-adjusted estimates and their precision (eg, 95% confidence interval). Make clear which confounders were adjusted for and why they were included	10
		(*b*) Report category boundaries when continuous variables were categorized	12
		(*c*) If relevant, consider translating estimates of relative risk into absolute risk for a meaningful time period	N/A
Other analyses	17	Report other analyses done—eg analyses of subgroups and interactions, and sensitivity analyses	13
**Discussion**			
Key results	18	Summarise key results with reference to study objectives	14
**Limitations**			
Interpretation	20	Give a cautious overall interpretation of results considering objectives, limitations, multiplicity of analyses, results from similar studies, and other relevant evidence	14-16
Generalisability	21	Discuss the generalisability (external validity) of the study results	18-19
**Other information**			
Funding	22	Give the source of funding and the role of the funders for the present study and, if applicable, for the original study on which the present article is based	21

*Give information separately for cases and controls in case-control studies and, if applicable, for exposed and unexposed groups in cohort and cross-sectional studies.

Note: An Explanation and Elaboration article discusses each checklist item and gives methodological background and published examples of transparent reporting. The STROBE checklist is best used in conjunction with this article (freely available on the Web sites of PLoS Medicine at http://www.plosmedicine.org/, Annals of Internal Medicine at http://www.annals.org/, and Epidemiology at http://www.epidem.com/). Information on the STROBE Initiative is available at www.strobe-statement.org.

For peer review only

The economic impact of childhood acute gastroenteritis on Malawian families and the healthcare system: a prospective cohort study

Journal:	*BMJ Open*
Manuscript ID	bmjopen-2017-017347.R1
Article Type:	Research
Date Submitted by the Author:	26-Jun-2017
Complete List of Authors:	Hendrix, Nathaniel; University of Washington, Pharmaceutical Outcomes Research and Policy Program<br>Bar-Zeev, Naor; University of Liverpool, The Centre for Global Vaccine Research, Institute of Infection & Global Health; University of Malawi, Malawi-Liverpool-Wellcome Trust Clinical Research Programme, College of Medicine<br>Atherly, Deborah; PATH<br>Chikafa, Jean; Malawi-Liverpool-Wellcome Trust Clinical Research Programme, College of Medicine, University of Malawi<br>Mvula, Hazzie; Karonga Prevention Study; London School of Hygiene and Tropical Medicine Department of Infectious Disease Epidemiology<br>Wachepa, Richard; Malawi-Liverpool-Wellcome Trust Clinical Research Programme, College of Medicine, University of Malawi<br>Crampin, Amelia; London School of Hygiene and Tropical Medicine Department of Infectious Disease Epidemiology<br>Mhango, Themba; Queen Elizabeth Central Hospital<br>Mwansambo, Charles; Government of Malawi Ministry of Health,<br>Heyderman, Robert; University College London, Division of Infection and Immunity; University of Malawi College of Medicine, Malawi Liverpool Wellcome Trust Clinical Research Programme<br>French, Neil; The Centre for Global Vaccine Research, Institute of Infection & Global Health; Malawi-Liverpool-Wellcome Trust Clinical Research Programme, College of Medicine, University of Malawi<br>Cunliffe, Nigel; University of Liverpool, Clinical Infection, Microbiology and Immunology<br>Pecenka, Clint; PATH
**Primary Subject Heading**:	Global health
Secondary Subject Heading:	Gastroenterology and hepatology, Health economics, Paediatrics
Keywords:	Gastrointestinal infections < GASTROENTEROLOGY, Paediatric gastroenterology < GASTROENTEROLOGY, Health economics < HEALTH SERVICES ADMINISTRATION & MANAGEMENT, International health services < HEALTH SERVICES ADMINISTRATION & MANAGEMENT, Tropical medicine < INFECTIOUS DISEASES

For peer review only

The economic impact of childhood acute gastroenteritis on Malawian families and the healthcare system: a prospective cohort study

Authors: Nathaniel Hendrix[1,2], Naor Bar-Zeev[3,4], Deborah Atherly[1], Jean Chikafa[4], Hazzie Mvula[5,6], Richard Wachepa[4], Amelia C Crampin[5,6], Themba Mhango[7] , Charles Mwansambo[8],Robert S Heyderman[4,9], Neil French[3,4], Nigel A Cunliffe[3], Clint Pecenka[1], for the VacSurv Consortium

Affiliations:
1. PATH, Seattle, USA
2. Pharmaceutical Outcomes Research and Policy Program, University of Washington, Seattle, USA.
3. The Centre for Global Vaccine Research, Institute of Infection & Global Health, University of Liverpool, Liverpool UK
4. Malawi-Liverpool-Wellcome Trust Clinical Research Programme, College of Medicine, University of Malawi, Blantyre Malawi
5. Karonga Prevention Study, Chilumba Malawi
6. London School of Hygiene and Tropical Medicine, London UK
7. Queen Elizabeth Central Hospital, Blantyre, Malawi
8. Ministry of Health, Lilongwe, Malawi
9. Division of Infection & Immunity, University College London, London UK

Corresponding author: Clint Pecenka

Tel: +1-206-285-3500

Email: cpecenka@path.org

Abstract word count: 199; text word count: 3,529

Running title: "Impact of childhood gastroenteritis in Malawi"

Key words: Malawi, gastroenteritis, diarrheal disease, low-income countries, burden of disease, medical impoverishment

ABSTRACT

For peer review only - http://bmjopen.bmj.com/site/about/guidelines.xhtml

Objectives: This prospective cohort study sought to estimate health system and household costs for episodes of diarrheal illness in Malawi.

Setting: Data was collected in two Malawian settings: a rural health center in Chilumba, and an urban tertiary care hospital in Blantyre.

Participants: Children under five years of age presenting with diarrheal disease between January 1, 2013 and November 21, 2014 were eligible for inclusion. Illnesses attributed to other underlying causes were excluded, as were illnesses commencing more than two weeks prior to presentation. Complete data was collected on 514 cases at both the time of the initial visit to the participating healthcare facility and six weeks after discharge.

Primary and secondary outcome measures: The primary outcome measure was the total cost of an episode of illness. Costs to the health system were gathered from chart review (drugs and diagnostics) and actual hospital expenditure (staff and facility costs). Household costs, including lost income, were obtained by interview with the parents/guardians of patients.

Results: Total costs in 2014 US$ for rural inpatient, rural outpatient, urban inpatient and urban outpatient were $65.33, $8.89, $60.23, and $14.51, respectively (excluding lost income). Mean household contributions to these costs were 15.8%, 9.8%, 21.3%, and 50.6%.

Conclusion: This study found significant financial burden from childhood diarrheal disease to the healthcare system and to households. The latter face the risk of consequent impoverishment, as the study demonstrates how the costs of seeking treatment bring the

For peer review only - http://bmjopen.bmj.com/site/about/guidelines.xhtml

income of the majority of families in all income strata below the national poverty line in the month of illness.

Strengths and limitations

- We collected costs of illness directly from patient caregivers and clinic records rather than relying on model-derived estimates.
- Collection of information on lost income means that we were able to provide more holistic measures of the effect of illness on household economy.
- Only two clinics were sampled, so the conclusions' generalizability to other locations in Malawi is uncertain.
- Estimates of the contribution of previous visits to overall costs are likely to be confounded by selection bias, as the most severe patients are likely to have been seen before and to have higher costs.

For peer review only - http://bmjopen.bmj.com/site/about/guidelines.xhtml

INTRODUCTION

Diarrhea is a major cause of childhood illness and death. It is responsible for an estimated 9% of all under-five deaths globally[1], and in Malawi results in at least 3,000 under-5 deaths annually[2]. Although the rotavirus vaccine was introduced in 2012, incidence of rotavirus hospitalization remains high at 123 per 100,000 infants (60 per 100,000 children aged 1 to 4 years), and this pathogen causes the largest share of gastroenteritis-related deaths, accounting for 25%[3,4]. The toll of diarrheal disease can be mitigated by treatments such as oral rehydration solution, zinc and in some cases, antibiotics or hospital-based care. Vaccines also play an important role in mitigating the spread and severity of diarrheal disease. Rotavirus vaccines, for example, have been introduced in many countries, including Malawi, where they prevent infection and severe disease, and have resulted in substantial reductions in rotavirus-associated illness and death[5,6].

In addition to the burden of disease and death in children, treatment costs can cause significant financial strain to households and health care systems alike. In order to understand comprehensively the impact of diarrheal disease and the potential economic benefit of vaccines, one must examine the actual costs of care incurred by the health care system and by households.

Most economic evaluations have relied on model-derived cost estimates based on extant, retrospective data. Very few studies have prospectively collected direct individual patient-level costs of diarrheal illness at both inpatient and outpatient settings[7]. Even fewer have collected such data from health facilities and households, including direct and indirect medical costs and

For peer review only - http://bmjopen.bmj.com/site/about/guidelines.xhtml

income loss estimates[8]. Additionally, many studies lack data on household income that would permit examination of differential short-term financial effects of illness by income level.

In this post-hoc analysis of data collected for a cost-effectiveness analysis[9], we sought to provide insight into the cost of treating diarrheal disease from the health system and societal perspectives in the context of a low-income country where receipt of both doses of the rotavirus vaccine is near 90%[10]. In other low-income settings, geographic setting (urban/rural), treatment setting (inpatient/outpatient), household income, and duration of illness have consistently demonstrated correlation with the economic burden of diarrheal disease[7,11,12]. We sought to explore these factors that drive cost of illness in this sub-Saharan, low-income setting, as well as to leverage our capture of prior costs to examine the effects of multiple sessions of care on cost.

CONTEXT AND METHODS

Study area and population

This prospective cohort study was conducted in Malawi, a country of approximately 16 million people in southern Africa. Its per capita gross national income of US$350 places it 210th out of 216 countries and territories ranked by the World Bank[13]. Over 80% of its population is rural, with much of that population engaged in subsistence farming[14].

The study was conducted at two sites: Queen Elizabeth Central Hospital (QECH), a national referral hospital that provides both inpatient and outpatient care, and Chilumba Rural Hospital (CRH), which provides outpatient primary care for children under 5 and accepts inpatients,

For peer review only - http://bmjopen.bmj.com/site/about/guidelines.xhtml

although generally low acuity cases. QECH is located in Blantyre, a district of approximately 1.3 million in Malawi's Southern Region. It receives approximately 90,000 presentations per year, 25,000 of which are admitted to the inpatient facility. CRH is located in Karonga, a district of approximately 200,000 in the Northern Region. Chilumba hosts a demographic surveillance site with a population approaching 40,000[15]. It sees 7,500 outpatients per year, of which about 700 are admitted inpatient. Both sites provide basic inpatient and outpatient care free of charge and were purposively selected to be representative of the care provided at different levels of the health system[9].

Inclusion criteria

Eligible participants were children under five years of age living in Blantyre district or within the demographic surveillance population in Chilumba and presenting with diarrhea at either facility during the study period (January 1, 2013 to November 21, 2014). Illnesses attributed to other underlying causes were excluded, as were illnesses commencing more than two weeks prior to presentation. In addition to identifying participants at registration/triage, admission logs and electronic records (at QECH only) were reviewed for missing cases.

Data collection

After identifying each participant and obtaining consent from their caregiver, a trained research assistant administered a standard case report form to the caregiver. The questionnaire included demographic information, socioeconomic status, and the history of the child's illness prior to presentation at the facility, as well as all illness-associated costs incurred prior to presenting at the facility. Daily review of medical charts was undertaken during admission for

For peer review only - http://bmjopen.bmj.com/site/about/guidelines.xhtml

inpatients to gather information on drugs dispensed, and laboratory investigations or procedures performed. All participants' homes were visited by research staff six weeks post-discharge to evaluate whether any subsequent illness-related costs were incurred by the household.

Categorization of expenses

Pre-treatment costs included direct medical costs paid by the household for care sought prior to the date of the index visit, which we defined as the date of an outpatient visit at either facility or the first day of an inpatient stay. Since the health facilities at which previous care was sought were not study sites, costs to the health system were not included in the pre-index costs.

Costs of the index visit were divided into health system and household costs. Costs to the health system included drugs, laboratory investigations, staff, and facility costs such as laundry, kitchen, sanitation, and security. The cost of drugs was calculated based on acquisition costs for drugs used at the index visit, with the quantity of injectable drugs being rounded up to the nearest full vial size. Investigations performed were costed on charge per test, except for those performed as part of clinical research, which were excluded. Staff costs for inpatient visits used the combined daily salary of all staff present divided by the number of beds in the ward, then multiplied by the patient's length of stay in days; staff costs of emergency outpatient visits were calculated by dividing the combined daily salary of staff by the mean daily number of visits to the department. Facility costs were calculated by dividing the facility's mean daily cost for services and subsistence goods by the number of hospital beds, then multiplying by the

For peer review only - http://bmjopen.bmj.com/site/about/guidelines.xhtml

patient's length of stay. The cost of consumable goods in the clinic such as tubing for intravenous drips were not included. Household costs included transportation to the index visit for the patient, their main caretaker and any other household visitors during admission; direct medical costs (costs of consultations, drugs, and diagnostics not covered by the essential health package); lost income attributable to the episode of disease; and subsistence (food and shelter) during the visit. Lost income was calculated by dividing the self-reported monthly income by the number of days in the month, then multiplying by the number of days spent in care-taking for this episode of illness.

Ongoing costs were assessed at a follow-up visit to the patient's home six weeks after facility discharge. Costs related to any long-term disability caused by the episode were not included among these ongoing costs. Again, costs to the health system for further treatment could not be ascertained due to other sites' being outside the network of research sites.

All costs were collected in Malawi kwacha and converted to US dollars based on the mid-market exchange rate per the Malawi Reserve Bank on July 15, 2014.

Statistical analysis

The study's minimum sample size of 88 was calculated to provide an estimate of household costs with a margin of error ≤10% given a coefficient of variation of 0.5[9,16].

Income quintiles were calculated based on self-reported monthly income within the sample itself---that is to say that a household labelled as belonging in the highest income quintile is within the top 20% of household incomes in this sample. Using one-way ANOVA, we tested for

For peer review only - http://bmjopen.bmj.com/site/about/guidelines.xhtml

differences among income quintiles in mean overall household costs at each geographic and treatment setting.

To explore associations proposed in previous research between prior treatment and cost, we constructed a generalized linear model (GLM) of total costs and household costs with log link on gamma distributional family, adjusted for length of stay, geographic setting, and treatment setting. We used a chi-squared test to compare the proportion of patients with prior/ongoing costs in different settings, and a rank-sum test to assess differences in component costs between these groups. We also used rank-sum tests to assess the significance of differences in total (health system plus household) and household cost between geographic and treatment settings.

Ethics

Ethical approval was provided by the National Health Sciences Research Committee, Lilongwe, Malawi (1073), and by the Research Ethics Committee of the University of Liverpool, United Kingdom (000490). Written consent was obtained from the parents or guardians of participating children.

RESULTS

Participants

A total of 529 individuals participated in this study, although 15 were excluded due to incomplete data. The majority (389) were recruited at QECH in Blantyre and the remaining 130 participants were recruited at CRH in Karonga. Inpatient cases accounted for 69% (269/389) of

For peer review only

For peer review only - http://bmjopen.bmj.com/site/about/guidelines.xhtml

187 the cases at QECH while only 17% (22/130) of cases in Karonga were admitted, reflecting a more serious case mix at a much larger national referral level hospital in Blantyre.

Table 1: Patient characteristics

	Rural		Urban		Total
	Inpatient	Outpatient	Inpatient	Outpatient	
N (% of total)	22 (4%)	108 (21%)	269 (52%)	120 (23%)	519
Mean Vesikari score (sd)	6.0 (2.9)	2.6 (2.2)	11.1 (3.9)	2.0 (3.4)	7.0 (5.5)
Sex: Male, not recorded(%)	13 (59%), 0	67 (62%), 2 (2%)	159 (59%), 2 (1%)	62 (52%), 1 (1%)	301(58%), 5(1%)
Mean age, months (sd)	14.0 (12.7)	15.8 (11.3)	13.4 (7.4)	15.8 (11.8)	14.3 (9.3)
Median length of stay, days (IQR)	2 (2.5)	N/A	3 (2)	N/A	3 (2) [inpatients only]

Total costs for the episode of illness as well as costs by payer are reported in **Table 2**, sorted by geography and treatment setting. The least expensive visit type was a rural, outpatient visit with total costs averaging $13.57; rural, inpatient visits were the costliest, averaging $76.94. Rank-sum tests confirmed the significance of differences in both total and household costs between geographic and treatment settings ($p < 0.001$ in all cases). Across all settings, the health system bore an average of 76% of the costs for each episode of illness.

We performed a sensitivity analysis to assess the potential impact to overall costs at the index visit if the 15 dropped observations were included. Were these costs representative of the 25th percentile of costs, mean overall costs would decrease from $39.15 to $38.32; were they in the 75th percentile of costs, mean overall costs would rise to $39.44. This would result in a 2% decrease in total costs, or a 0.6% increase, respectively, compared to the weighted average of costs across all visit types.

For peer review only - http://bmjopen.bmj.com/site/about/guidelines.xhtml

Table 2: Mean (SD) total direct and indirect costs for the episode of illness by geography and treatment setting

	Rural		Urban	
	Inpatient	Outpatient	Inpatient	Outpatient
Health system costs	$55.04 ($26.82)	$8.02 ($2.90)	$47.21 ($56.69)	$7.18 ($4.05)
Household costs	$19.16 ($19.30)	$1.81 ($2.58)	$25.36 ($20.47)	$15.48 ($20.75)
Total costs	$76.94 ($32.61)	$13.57 ($4.70)	$73.78 ($51.39)	$23.13 ($21.68)

For inpatient settings, staff and facility costs contribute the most to health system costs (see **Table 3**). These two categories represent between 60 and 80% of total costs across geographic setting. Laboratory investigations dominate outpatient costs, representing roughly 60% in both urban and rural outpatient settings.

Table 3: Mean (SD) costs to health system for index visit by category

	Rural		Urban	
	Inpatient	Outpatient	Inpatient	Outpatient
Drugs	$4.46 ($3.94)	$1.45 ($2.53)	$9.40 ($20.29)	$1.19 ($2.16)
Labs	$5.39 ($1.07)	$4.96 ($1.17)	$7.60 ($5.21)	$4.73 ($3.36)
Staff	$17.03 ($10.37)	$1.61 ($0)	$21.47 ($30.51)	$1.26 ($0)
Facilities	$28.35 ($17.26)	$0 ($0)	$8.73 ($12.41)	$0 ($0)
Total	$55.04 ($26.82)	$8.02 ($2.90)	$47.21 ($56.69)	$7.18 ($4.05)

Lost income and direct medical expenses make up the majority of household costs for all except rural, outpatient visits, whose costs are dominated by transportation and lost income (see

For peer review only

For peer review only - http://bmjopen.bmj.com/site/about/guidelines.xhtml

Table 4). Of patients seen at the urban facility, 35% had sought treatment prior to their index visit, compared to 2% of patients seen at the rural facility ($p < 0.001$). On the other hand, 13% of patients in Karonga had ongoing costs after the index visit, compared to 2% of patients seen at the Blantyre site ($p < 0.001$).

Table 4: Mean (SD) household costs for an episode of illness

	Rural		Urban		Total
	Inpatient	Outpatient	Inpatient	Outpatient	
Direct medical expenses prior to index visit	$0.21 ($0.72)	$0.08 ($0.58)	$0.98 ($3.92)	$0.71 ($1.55)	$0.77 ($3.13)
Direct medical expenses of index visit	$8.61 ($9.62)	$0.24 ($0.68)	$10.69 ($10.66)	$5.85 ($10.21)	$7.31 ($10.17)
Transportation to and subsistence at index visit	$4.38 ($5.28)	$0.51 ($0.54)	$2.68 ($3.00)	$0.45 ($1.91)	$2.04 ($2.83)
Lost income due to index visit	$4.13 ($9.62)	$0.50 ($0.68)	$8.06 ($10.66)	$6.22 ($10.22)	$6.36 ($10.17)
Direct medical expenses after index visit	$0.87 ($2.10)	$0.38 ($1.66)	$0.21 ($2.41)	$0.16 ($1.19)	$0.26 ($2.02)
Total household costs	$19.16 ($19.30)	$1.81 ($2.58)	$25.36 ($20.47)	$15.48 ($20.75)	$19.32 ($20.66)

Using one-way ANOVA to detect differences in mean household expenditure (exclusive of lost income) between income quintiles, we found a significant difference only in urban, inpatient encounters (see **Figure 1**). The highest income level in this group (> $79.50 per month) had higher expenditures than any other income group, with the difference between this group and the lowest income group (< $19.32 per month) being especially noticeable. The ANOVA test

For peer review only

For peer review only - http://bmjopen.bmj.com/site/about/guidelines.xhtml

showed no significant differences for any other combination of geographic and treatment settings.

Costs to the households of patients for whom previous treatment was sought were 2.04 times higher ($p < 0.001$) once adjusted for length of stay, geography and treatment setting; overall costs were 1.25 times higher ($p = 0.016$). This difference in overall costs is driven in part by spending prior to the index visit, which averaged between $0.92 for rural, outpatient index visits to $3.01 for rural, inpatient index visits. A rank-sum test on transportation costs by prior treatment status showed significantly higher transportation costs for this group ($p < 0.001$), suggesting the source of these differences. Median monthly income for the group who sought prior treatment was higher than the group who did not, at $34.09 and $22.73, respectively. A rank-sum test again showed a significant difference in both monthly income and lost income due to the illness (length of stay was not significantly different between the groups). We repeated the rank-sum test looking for variation in transportation costs during the rainy seasons (November through April) of those who received prior treatment, but this test's results were not significant.

DISCUSSION

Even following the introduction of the rotavirus vaccine, diarrheal disease continues to be a significant burden on the world and on low-income countries in particular. While deaths among African children due to diarrhea declined 43% between 2000 and 2013[17], the number of cases remains high, with one study estimating 3.3 episodes of diarrhea annually per child in Africa[18].

For peer review only - http://bmjopen.bmj.com/site/about/guidelines.xhtml

This study focused on the economic burden of these diseases by combining costs ascertained through both interviews and chart review. It found significant household costs primarily in the inpatient and urban settings with lost income and direct medical costs dominating the contributing costs. Even rural, outpatient visits can be financially problematic, however, if they are frequent, or if the family is especially vulnerable. Household costs for the episode of illness (excluding lost income) exceeded monthly household income in 8% of cases treated inpatient and 3% of cases treated outpatient; including lost income, these numbers rise to 17% and 9%, respectively.

Taking into account lost income, mean household costs for patients seen in the rural health center were $19.16 and $1.81 for inpatient and outpatient treatment, while patients seen at the urban hospital had mean household costs of $25.36 and $15.48 for inpatient and outpatient treatment. The high cost of inpatient treatment in the urban setting likely reflects the higher acuity of illness at that setting: the mean Vesikari score of study participants seen in QECH's inpatient ward was 11, which indicates severe disease. Rural inpatients, by comparison, had a mean Vesikari score of 6.

Putting household expenditures in context, 72.2% of the Malawian population lives on less than $1.25 per person per day[19]. Even an outpatient visit in a rural setting, then, is likely to impose costs exceeding a day's income for this population, while a costlier inpatient visit is likely to account for almost 3 weeks' income. The equivalents for a US household would be to face costs of $147 and $3,095, respectively[20]. It should be noted, though, that in a low-income setting, a much larger percent of income goes to core goods such as food and shelter: as such, it is more

For peer review only - http://bmjopen.bmj.com/site/about/guidelines.xhtml

difficult to offset health-related costs. Having savings adequate to cover medical costs is also less common in this setting, where, although 76% report saving when possible, 90% of households say that they sometimes run out of money for basic goods[21]. This can bring about debt and reduced expenditure on clothing, education, shelter, and other similar goods.

The results of our study can be compared with several other studies of the economic burden of diarrheal disease in the low-income, sub-Saharan context. An analysis of economic data from the Global Multicenter Enteric Study found mean household costs of $2.77, $6.57, and $4.33 (in 2014 USD) for Gambia, Kenya, and Mali, respectively, for a representative sample of inpatient and outpatient medical encounters, not including lost income[22]. A Rwandan study of inpatient admissions for childhood gastroenteritis found household costs of $66.08 (including lost income) and costs to the medical system of $34.83[23]. Finally, a study from Ghana found costs to the medical system of $5.14 for outpatient visits and $122.07 for inpatient visits[8].

When costs are analyzed by income quintile, mean household expenditure by quintile is only significantly different in the urban, inpatient setting. This demonstrates that the poorest segments of Malawian society who attend public healthcare facilities generally incur the same costs as their wealthier peers in the same settings. This finding is supported by other evidence from Malawi which found that care-seeking behavior for children with diarrhea does not vary by income level[24].

The pattern of identical expenditure across income quintiles in most settings means that the poorest households expose themselves to greater risk of impoverishment by seeking treatment (see **Figure 2**). After accounting for the costs of the episode of illness, no household in the

For peer review only - http://bmjopen.bmj.com/site/about/guidelines.xhtml

lowest income quintile of our sample was left with more than $0.60 of income per day for the month in which the illness occurred—well below the government's definition of "ultra-poverty" as $1.74 per household per day[25]. Furthermore, the costs of the episode of illness exceeded the monthly income of 27% of households in this group. Even among the highest income quintile of our sample, 19% of households were brought temporarily below the poverty line of $2.80 per day by the costs of the episode of illness.

We are left to explain why spending increases with income quintile in the urban inpatient setting (see **Figure 1**). One explanation may be that price sensitivity may play a role in care decisions in the urban inpatient setting. If this were the case, the children of poorer Malawians may not receive the full extent of care that they need due to concerns about the costs associated with seeking treatment, even when the treatment itself is free. Mean total household costs were the highest for this setting, and the patients' caretakers must balance the risks of expenses that they cannot afford with the risk that their child's illness will worsen when they choose to seek or not seek care. Lower income families may, rationally, apply a different threshold for seeking treatment than higher income families. Another possibility may be a tendency of higher income families to bring more visitors or to buy higher cost subsistence goods. In order to test this possibility, we ran an ANOVA test on mean spending by income quintile on visitors, transportation, and shelter costs (both together and separately), but did not find significant differences. Instead, the same test shows significantly increased mean spending on direct medical expenses as income quintile increases.

For peer review only - http://bmjopen.bmj.com/site/about/guidelines.xhtml

One driver of high overall costs in the urban setting at large may be the seeking of prior care. Of patients seen in the urban setting, 35% had been seen at a prior healthcare facility. Testing the relationship between prior care and income quintile using a logistic regression does not suggest a significant relationship between the two, whether income quintile is represented as a continuous or factor variable. Excluding lost wages, household costs for cases in which prior care is sought cost twice as much as cases in which the index visit is the first encounter with the healthcare system during this episode of illness. Transportation costs are the likely source of difference, suggesting that patients who sought treatment prior to the index visit come to the hospital either from farther away or more urgently than those for whom the index visit was their first visit related to this episode. Transportation costs predominate costs accrued prior to the index visit as well, accounting for 62%; spending at health centers and pharmacies accounted for 13% and 11%, respectively. Selection bias likely plays a role as well, as the most seriously ill patients are likely both to be seen multiple times and to incur greater overall costs.

The conclusions in this study are limited by its being a post-hoc, unpowered analysis of data gathered for a cost-effectiveness study. That is, the sample size of the study was not powered to detect differences between treatment settings, geography, or any of the other characteristics examined in our regression analysis. The study's generalizability may also be limited due to the data collection's having taken place in two locations that represent extremes of acuity and population density within the Malawian healthcare system. We cannot rule out greater geographic variation in financial outcomes than our study results suggest.

For peer review only - http://bmjopen.bmj.com/site/about/guidelines.xhtml

Future work in this area should focus on the long-term consequences to the household of financial shock associated with an episode of diarrheal disease, or with repeated diarrhea due to undernourishment and other risk factors. Especially of interest is the long-term loss of economic potential due to childhood illness: if a child is frequently or severely ill, health-related expenditures, including time needed for caretaking, may preclude investment in other areas that could improve the financial situation of a household.

Conclusion

This study has evaluated directly the cost of 514 episodes of childhood diarrheal disease to both the households and health system of Malawi. It found that households bear a substantial fraction of the total costs of an episode of illness and that there is a considerable risk of impoverishment due to severe or repeated episodes of diarrheal disease.

The conclusions of this study are broadly applicable in the sub-Saharan setting—especially to those countries where the rotavirus vaccine is widely used—regardless of whether healthcare is provided to the public for free or through an insurance program. In the latter case, only direct medical costs to the household would be covered. Lost income, transportation, and ongoing costs of care were all found in this study to impose substantial financial burdens, and this observation would not be changed by the use of health insurance.

This study's findings can be applied to cost-effectiveness analyses of enteric vaccines and other means of preventing the transmission of diarrheal disease and can be used by healthcare planners to help prioritize interventions. By providing data on the lost financial opportunity

For peer review only

For peer review only - http://bmjopen.bmj.com/site/about/guidelines.xhtml

345 caused by an episode of diarrheal disease, this study allows for a more precise accounting of
346 how an averted episode of diarrheal disease should be valued.

For peer review only

For peer review only - http://bmjopen.bmj.com/site/about/guidelines.xhtml

Conflict of interest: NBZ, NAC and NF have received investigator-initiated research grants from GSK; and NBZ and NAC from Takeda Pharmaceuticals. All other authors have no conflicts to declare.

Contributorship statement: NB-Z, DA, and NAC designed the study with input from AC, RSH, and NF. NB-Z, JC, HM, RW, CM, and TM obtained the data. NH, NB-Z, CP, and DA designed and performed the data analysis. NH wrote the first draft of the manuscript. All listed authors contributed to the interpretation of the data, reviewed the work prior to submission, and have agreed to be responsible for its content.

Data sharing statement: Data collected as part of this study is not available due to its not having been included in the approved protocol.

Funding: This work was supported by Wellcome Trust Programme Grant [WT091909], PATH and the Bill & Melinda Gates Foundation [OPP1053539].

Preliminary results from this study were presented at the 12th International Rotavirus Symposium in Melbourne, Victoria.

Corresponding author: Clint Pecenka
Tel: +1-206-285-3500
Email: cpecenka@path.org

Members of the VacSurv Consortium:

For peer review only

James Beard (London School of Hygiene and Tropical Medicine [LSHTM], London, UK); Anthony Costello (World Health Organization, Geneva Switzerland; formerly University College London [UCL], London, UK); Miren Iturriza-Gomara (University of Liverpool [UoL], Liverpool, UK); Khuzwayo Jere (UoL) Carina King (UCL, London, UK); Sonia Lewycka (University of Auckland, Auckland, New Zealand; formerly UCL); Osamu Nakagomi (Nagasaki University, Japan); Umesh Parashar (Centers for Disease Control & Prevention [CDC], Atlanta, GA, USA); Tambosi Phiri (Mai Mwana Project, Mchinji, Malawi); Jacqueline E Tate (CDC) Atlanta, GA, USA; Jennifer R Verani (CDC) Atlanta, GA, USA; Cynthia G Whitney (CDC) Atlanta, GA, USA.

References

1. Liu L, Johnson H, Cousens S. Global, regional, and national causes of child mortality in 2000-2010: an updated systematic analysis. Under Revis with Lancet [Internet]. Elsevier Ltd; **2012**; 6736(14). Available from: http://dx.doi.org/10.1016/S0140-6736(14)61698-6

2. World Health Organization. Global Health Observatory Data Repository [Internet]. WHO. World Health Organization; 2016 [cited 2016 Aug 3]. Available from: http://www.who.int/gho/database/en/

3. Institute for Health Metrics and Evaluation. The Global Burden of Disease: Generating Evidence, Guiding Policy [Internet]. Seattle, WA; 2013. Available from: http://www.healthdata.org/policy-report/global-burden-disease-generating-evidence-guiding-policy

For peer review only - http://bmjopen.bmj.com/site/about/guidelines.xhtml

4. Bar-Zeev N, Jere KC, Bennett A, et al. Population Impact and Effectiveness of Monovalent Rotavirus Vaccination in Urban Malawian Children 3 Years after Vaccine Introduction: Ecological and Case-Control Analyses. Clin Infect Dis. **2016**; 62(Suppl 2):S213–S219.

5. Bar-Zeev N, Kapanda L, Tate JE, et al. Effectiveness of a monovalent rotavirus vaccine in infants in Malawi after programmatic roll-out: an observational and case-control study. Lancet Infect Dis [Internet]. **2015** [cited 2016 Nov 5]; 15(4):422–428. Available from: http://linkinghub.elsevier.com/retrieve/pii/S1473309914710606

6. Groome MJ, Page N, Cortese MM, et al. Effectiveness of monovalent human rotavirus vaccine against admission to hospital for acute rotavirus diarrhoea in South African children: a case-control study. Lancet Infect Dis [Internet]. **2014** [cited 2016 Nov 5]; 14(11):1096–1104. Available from: http://linkinghub.elsevier.com/retrieve/pii/S1473309914709405

7. Rheingans R, Kukla M, Faruque ASG, et al. Determinants of household costs associated with childhood diarrhea in 3 South Asian settings. Clin Infect Dis [Internet]. **2012** [cited 2016 Aug 3]; 55 Suppl 4:S327-35. Available from: http://www.ncbi.nlm.nih.gov/pubmed/23169945

8. Aikins M, Armah G, Akazili J, Hodgson A. Hospital health care cost of diarrheal disease in Northern Ghana. J Infect Dis [Internet]. **2010** [cited 2016 Aug 3]; 202 Suppl:S126-30. Available from: http://www.ncbi.nlm.nih.gov/pubmed/20684692

9. Bar-Zeev N, Tate JE, Pecenka C, et al. Cost-Effectiveness of Monovalent Rotavirus

For peer review only

Vaccination of Infants in Malawi: A Postintroduction Analysis Using Individual Patient–Level Costing Data. Clin Infect Dis [Internet]. **2016** [cited 2016 Nov 5]; 62(suppl 2):S220–S228. Available from: http://cid.oxfordjournals.org/lookup/doi/10.1093/cid/civ1025

10. Bar-Zeev N, Kapanda L, Tate JE, et al. Effectiveness of a monovalent rotavirus vaccine in infants in Malawi after programmatic roll-out: an observational and case-control study. Lancet Infect Dis [Internet]. **2015** [cited 2016 Nov 5]; 15(4):422–8. Available from: http://www.ncbi.nlm.nih.gov/pubmed/25638521

11. Burke RM, Rebolledo PA, Embrey SR, et al. The burden of pediatric diarrhea: a cross-sectional study of incurred costs and perceptions of cost among Bolivian families. BMC Public Health [Internet]. BioMed Central; **2013** [cited 2016 Dec 2]; 13(1):708. Available from: http://bmcpublichealth.biomedcentral.com/articles/10.1186/1471-2458-13-708

12. MacIntyre UE, Villiers FPR de. The economic burden of diarrheal disease in a tertiary level hospital, Gauteng, South Africa. J Infect Dis [Internet]. Oxford University Press; **2010** [cited 2016 Dec 2]; 202 Suppl(Supplement 1):S116-25. Available from: http://www.ncbi.nlm.nih.gov/pubmed/20684690

13. World Bank. Gross National Income Per Capita 2015 (Atlas Method) [Internet]. [cited 2016 Nov 5]. Available from: http://databank.worldbank.org/data/download/GNIPC.pdf

14. National Statistical Office [Malawi], ICF International. Malawi Demographic and Health Survey. Zomba, Malawi and Rockville, Maryland, USA; 2015.

15. Crampin AC, Dube A, Mboma S, et al. Profile: the Karonga Health and Demographic

For peer review only - http://bmjopen.bmj.com/site/about/guidelines.xhtml

426 Surveillance System. Int J Epidemiol [Internet]. **2012** [cited 2016 Aug 3]; 41(3):676–85.
427 Available from: http://www.ncbi.nlm.nih.gov/pubmed/22729235

428 16. World Health Organization. Guidelines for estimating the economic burden of diarrhoeal
429 disease with focus on assessing the costs of rotavirus diarrhoea. Geneva; 2005.

430 17. Liu L, Oza S, Hogan D, et al. Global, regional, and national causes of child mortality in
431 2000–13, with projections to inform post-2015 priorities: an updated systematic analysis.
432 Lancet. **2015**; 385(9966):430–440.

433 18. Fischer Walker CL, Perin J, Aryee MJ, et al. Diarrhea incidence in low- and middle-income
434 countries in 1990 and 2010: a systematic review. BMC Public Health [Internet]. BioMed
435 Central; **2012** [cited 2016 Aug 3]; 12(1):220. Available from:
436 http://bmcpublichealth.biomedcentral.com/articles/10.1186/1471-2458-12-220

437 19. United Nations Development Programme. Global 2015 Human Development Report.
438 New York, New York, USA; 2015.

439 20. US. Bureau of the Census, Real Median Household Income in the United States
440 [MEHOINUSA672N] [Internet]. [cited 2016 Aug 28]. Available from:
441 https://fred.stlouisfed.org/series/MEHOINUSA672N

442 21. Reserve Bank of Malawi. Malawi: Baseline Financial Literacy and Consumer Protection
443 Household Survey. Zomba, Malawi; 2014.

444 22. Rheingans R, Kukla M, Adegbola RA, et al. Exploring household economic impacts of
445 childhood diarrheal illnesses in 3 African settings. Clin Infect Dis [Internet]. Oxford

For peer review only

University Press; **2012** [cited 2017 Jan 1]; 55 Suppl 4(Suppl 4):S317-26. Available from: http://www.ncbi.nlm.nih.gov/pubmed/23169944

23. Ngabo F, Mvundura M, Gazley L, et al. The Economic Burden Attributable to a Child's Inpatient Admission for Diarrheal Disease in Rwanda. Griffiths UK, editor. PLoS One [Internet]. Public Library of Science; **2016** [cited 2017 Jan 1]; 11(2):e0149805. Available from: http://dx.plos.org/10.1371/journal.pone.0149805

24. National Statistical Office (NSO) [Malawi] and ICF International. Malawi Demographic and Health Survey 2015-16: Key Indicators Report. Zomba, Malawi, and Rockville, Maryland, USA; 2016.

25. National Statistical Office (NSO) [Malawi]. Integrated Household Survey, 2010-2011. Zomba, Malawi; 2012.

For peer review only

Figure 1: Mean total household cost (not including lost income) of inpatient, urban index visit by income quintile

203x127mm (300 x 300 DPI)

view only

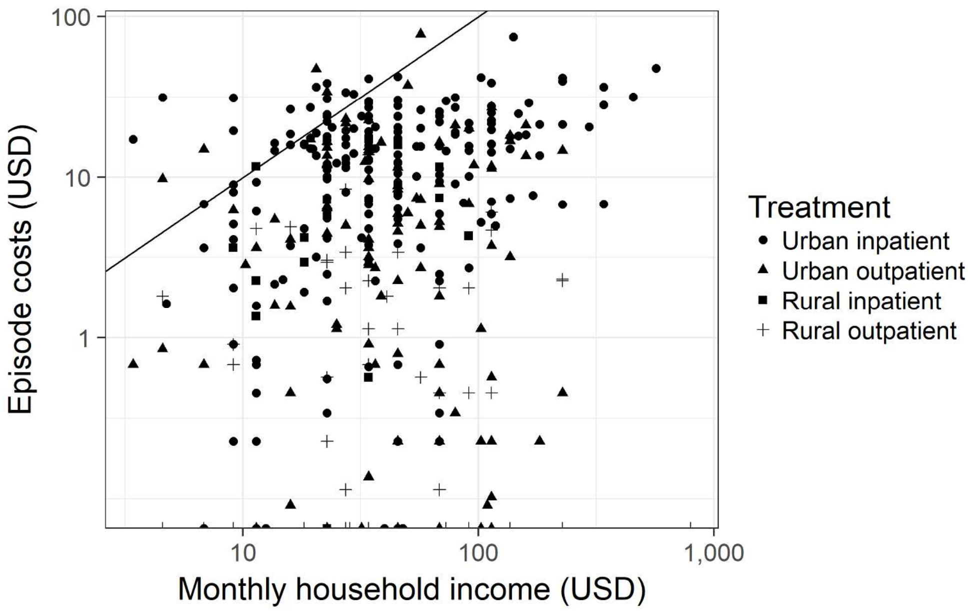

Figure 2: Log monthly household income versus log costs for the episode of illness, not including lost income. The line represents episode costs equal to monthly income.

203x127mm (300 x 300 DPI)

For peer review only

STROBE 2007 (v4) Statement—Checklist of items that should be included in reports of *cohort studies*

Section/Topic	Item #	Recommendation	Reported on page #
**Title and abstract**	1	(*a*) Indicate the study's design with a commonly used term in the title or the abstract	2
		(*b*) Provide in the abstract an informative and balanced summary of what was done and what was found	2
**Introduction**			
Background/rationale	2	Explain the scientific background and rationale for the investigation being reported	4
Objectives	3	State specific objectives, including any prespecified hypotheses	4-5
**Methods**			
Study design	4	Present key elements of study design early in the paper	5
Setting	5	Describe the setting, locations, and relevant dates, including periods of recruitment, exposure, follow-up, and data collection	5-6
Participants	6	(*a*) Give the eligibility criteria, and the sources and methods of selection of participants. Describe methods of follow-up	5-6
		(*b*) For matched studies, give matching criteria and number of exposed and unexposed	N/A
Variables	7	Clearly define all outcomes, exposures, predictors, potential confounders, and effect modifiers. Give diagnostic criteria, if applicable	5-8
Data sources/ measurement	8*	For each variable of interest, give sources of data and details of methods of assessment (measurement). Describe comparability of assessment methods if there is more than one group	7-8
Bias	9	Describe any efforts to address potential sources of bias	17-18
Study size	10	Explain how the study size was arrived at	8
Quantitative variables	11	Explain how quantitative variables were handled in the analyses. If applicable, describe which groupings were chosen and why	6-8
Statistical methods	12	(*a*) Describe all statistical methods, including those used to control for confounding	8-9
		(*b*) Describe any methods used to examine subgroups and interactions	8-9
		(*c*) Explain how missing data were addressed	9-10
		(*d*) If applicable, explain how loss to follow-up was addressed	N/A
		(*e*) Describe any sensitivity analyses	10
**Results**			

For peer review only

Participants	13*	(a) Report numbers of individuals at each stage of study—eg numbers potentially eligible, examined for eligibility, confirmed eligible, included in the study, completing follow-up, and analysed	9
		(b) Give reasons for non-participation at each stage	N/A
		(c) Consider use of a flow diagram	N/A
Descriptive data	14*	(a) Give characteristics of study participants (eg demographic, clinical, social) and information on exposures and potential confounders	9-11
		(b) Indicate number of participants with missing data for each variable of interest	9
		(c) Summarise follow-up time (eg, average and total amount)	6
Outcome data	15*	Report numbers of outcome events or summary measures over time	10
Main results	16	(*a*) Give unadjusted estimates and, if applicable, confounder-adjusted estimates and their precision (eg, 95% confidence interval). Make clear which confounders were adjusted for and why they were included	10
		(*b*) Report category boundaries when continuous variables were categorized	12
		(*c*) If relevant, consider translating estimates of relative risk into absolute risk for a meaningful time period	N/A
Other analyses	17	Report other analyses done—eg analyses of subgroups and interactions, and sensitivity analyses	13
**Discussion**			
Key results	18	Summarise key results with reference to study objectives	14
**Limitations**			
Interpretation	20	Give a cautious overall interpretation of results considering objectives, limitations, multiplicity of analyses, results from similar studies, and other relevant evidence	14-16
Generalisability	21	Discuss the generalisability (external validity) of the study results	18-19
**Other information**			
Funding	22	Give the source of funding and the role of the funders for the present study and, if applicable, for the original study on which the present article is based	21

*Give information separately for cases and controls in case-control studies and, if applicable, for exposed and unexposed groups in cohort and cross-sectional studies.

Note: An Explanation and Elaboration article discusses each checklist item and gives methodological background and published examples of transparent reporting. The STROBE checklist is best used in conjunction with this article (freely available on the Web sites of PLoS Medicine at http://www.plosmedicine.org/, Annals of Internal Medicine at http://www.annals.org/, and Epidemiology at http://www.epidem.com/). Information on the STROBE Initiative is available at www.strobe-statement.org.

For peer review only

STROBE 2007 (v4) Statement—Checklist of items that should be included in reports of *cohort studies*

Section/Topic	Item #	Recommendation	Reported on page #
**Title and abstract**	1	(*a*) Indicate the study's design with a commonly used term in the title or the abstract	2
		(*b*) Provide in the abstract an informative and balanced summary of what was done and what was found	2
**Introduction**			
Background/rationale	2	Explain the scientific background and rationale for the investigation being reported	4
Objectives	3	State specific objectives, including any prespecified hypotheses	4
**Methods**			
Study design	4	Present key elements of study design early in the paper	5
Setting	5	Describe the setting, locations, and relevant dates, including periods of recruitment, exposure, follow-up, and data collection	5
Participants	6	(*a*) Give the eligibility criteria, and the sources and methods of selection of participants. Describe methods of follow-up	5
		(*b*) For matched studies, give matching criteria and number of exposed and unexposed	N/A
Variables	7	Clearly define all outcomes, exposures, predictors, potential confounders, and effect modifiers. Give diagnostic criteria, if applicable	5
Data sources/ measurement	8*	For each variable of interest, give sources of data and details of methods of assessment (measurement). Describe comparability of assessment methods if there is more than one group	7
Bias	9	Describe any efforts to address potential sources of bias	17
Study size	10	Explain how the study size was arrived at	8
Quantitative variables	11	Explain how quantitative variables were handled in the analyses. If applicable, describe which groupings were chosen and why	6
Statistical methods	12	(*a*) Describe all statistical methods, including those used to control for confounding	8
		(*b*) Describe any methods used to examine subgroups and interactions	8
		(*c*) Explain how missing data were addressed	9
		(*d*) If applicable, explain how loss to follow-up was addressed	N/A
		(*e*) Describe any sensitivity analyses	10
**Results**			

For peer review only

Participants	13*	(a) Report numbers of individuals at each stage of study—eg numbers potentially eligible, examined for eligibility, confirmed eligible, included in the study, completing follow-up, and analysed	9
		(b) Give reasons for non-participation at each stage	9
		(c) Consider use of a flow diagram	N/A
Descriptive data	14*	(a) Give characteristics of study participants (eg demographic, clinical, social) and information on exposures and potential confounders	9
		(b) Indicate number of participants with missing data for each variable of interest	9
		(c) Summarise follow-up time (eg, average and total amount)	6
Outcome data	15*	Report numbers of outcome events or summary measures over time	10
Main results	16	(*a*) Give unadjusted estimates and, if applicable, confounder-adjusted estimates and their precision (eg, 95% confidence interval). Make clear which confounders were adjusted for and why they were included	10
		(*b*) Report category boundaries when continuous variables were categorized	12
		(*c*) If relevant, consider translating estimates of relative risk into absolute risk for a meaningful time period	N/A
Other analyses	17	Report other analyses done—eg analyses of subgroups and interactions, and sensitivity analyses	12
**Discussion**			
Key results	18	Summarise key results with reference to study objectives	14
**Limitations**			
Interpretation	20	Give a cautious overall interpretation of results considering objectives, limitations, multiplicity of analyses, results from similar studies, and other relevant evidence	14
Generalisability	21	Discuss the generalisability (external validity) of the study results	18
**Other information**			
Funding	22	Give the source of funding and the role of the funders for the present study and, if applicable, for the original study on which the present article is based	19

*Give information separately for cases and controls in case-control studies and, if applicable, for exposed and unexposed groups in cohort and cross-sectional studies.

Note: An Explanation and Elaboration article discusses each checklist item and gives methodological background and published examples of transparent reporting. The STROBE checklist is best used in conjunction with this article (freely available on the Web sites of PLoS Medicine at http://www.plosmedicine.org/, Annals of Internal Medicine at http://www.annals.org/, and Epidemiology at http://www.epidem.com/). Information on the STROBE Initiative is available at www.strobe-statement.org.

For peer review only
